# Scoping Review on the Use of Music for Emotion Regulation

**DOI:** 10.3390/bs14090793

**Published:** 2024-09-09

**Authors:** Hyun Ju Chong, Hyeon Joo Kim, Bohyun Kim

**Affiliations:** 1 Music Therapy Department of Graduate School, Ewha Womans University, Seoul 03760, Republic of Korea; hju@ewha.ac.kr (H.J.C.); hyeonjookim@ewhain.net (H.J.K.); 2 Ewha Music Wellness Research Center, Seoul 03765, Republic of Korea

**Keywords:** music, emotion, regulation, regulation processes, scoping review

## Abstract

With increasing interest in the emotional responses to music, some studies are specifically looking into music’s regulatory function on emotion, known as “music emotion regulation (MER)”. The purpose of this study was to examine the concepts on the regulatory impact of music on emotion using a scoping review procedure. Through an electronic database and manual searches based on the guidelines suggested by the JBI Manual for Evidence Synthesis, a total of 47 studies were identified and included for analysis. The results showed that there were some definitional challenges in each component of music emotion regulation. Most studies treated music as a single variable without exploring the regulatory mechanism of the intra-musical elements that constitute music. When examining the regulatory impact of music on emotion, emotion was inclusive of other terms ranging from feeling to mood. Most of the MER studies employed the terms used in the emotion regulation strategies; however, there were some regulatory processes that pertained solely to music’s cathartic effect. Overall, the results showed that music emotion regulation (MER) was not clearly distinguished from music emotion (ME) studies. Future research should allocate more attention to the theoretical mechanism behind music’s intrinsic regulatory effect in emotion regulation (MER) studies.

## 1. Introduction

Musical behavior is a profound facet of human expression that encapsulates one’s thoughts, emotions, and feelings. By engaging with music as an auditory medium, individuals can communicate and connect with others, experiencing a diverse spectrum of emotions and sentiments. Among various levels of music engagement, receptive music allows listeners to explore emotions triggered by music. The receptive aspect of musical experience enables individuals to grasp and interpret the emotional subtleties conveyed by musical elements, including rhythm, melody, harmony, and lyrics [1].

For decades, researchers have been intrigued by the impact of music on human emotions and its role in regulating them. It is abundantly clear that music serves as a conduit for emotions, prompting investigations into various factors that mediate the relationship between human emotion and music. These factors encompass direct cues, such as psychophysical cues or expectancy mechanisms within the music itself, as well as indirect or subjective sources, such as personal memories and associations [2,3,4].

Over the years, research into the emotional aspects of music has witnessed significant growth, with active exploration dating back to the 1930s [5]. While music is primarily an auditory stimulus perceived through hearing and listening, its effects extend well beyond the auditory domain, evoking multisensory responses at perceptual, cognitive, somatic, and behavioral levels. Despite varying research outcomes that may either support or contradict each other, the evidence unequivocally points to music as a potent elicitor of emotions, driven by the listener’s physiological responses, which stem from the autonomic nervous system to emotional actions influenced by referential experiences. Therefore, when considering the effects of music, it encompasses a diverse range of emotional responses in terms of forms, traits, and dimensions.

Emotion regulation can be defined as purposeful processes aimed at influencing the intensity, duration, and nature of one’s emotional experiences [6]. The term “emotion regulation” is often used interchangeably with “affect regulation” and “mood regulation”. Gross [7] distinguished between emotion and mood based on their degree of behavioral expression and whether they are elicited by specific events. For instance, emotions are typically more behavioral in nature and are often tied to specific events that evoke those emotions. Consequently, the regulation of emotion or moods may target different aspects of emotional response, including physiological responses and behavioral expressions.

The term “regulation” refers to a process aimed at facilitating a transition from one state of being to a more manageable and comfortable one, making emotion regulation a central concept in the field of well-being [8]. However, reaching a comfortable emotional state can involve various pathways, whether it is uplifting the mood, grounding oneself in negative emotions temporarily, or suppressing the emotion [9]. These processes encompass both extrinsic and intrinsic mechanisms for monitoring and adjusting emotional reactions [10]. A meta-analysis of the emotion regulation study indicated that certain strategies—namely attention deployment, cognitive change, and response modulation—were sequentially effective [11].

Furthermore, scientific research has illuminated how music engages multiple regions of the brain, measuring brain activity patterns. With the development of technology, non-invasive neuroimaging devices such as an electroencephalogram (EEG), functional magnetic resonance imaging (fMRI), and functional near-infrared spectroscopy (fNIRS) are used to derive evidence-based effects of music during listening [12,13]. Among the studies using neuroimaging devices, fNIRS studies consistently showed that emotion may have an effect on the construal level, particularly affecting prefrontal cortex (PFC) activation related to the valence and intensity of motivation [14]. These advanced measurement devices are essential for music emotion regulation studies.

In terms of emotion regulation, listeners actively choose music that aligns with their desired emotional outcomes, seeking to shift their mood toward a positive state or alleviate negative emotions [15]. The emotional experience of music manifests across various dimensions, depending on whether the source of the emotional trigger is intrinsic to the music itself (implicit) or arises from the listener’s subjective associations with the music (explicit) [16].

When music is utilized for emotion regulation, it serves as both an explicit and implicit resource. For implicit regulation, music serves as a facilitator for an individual’s emotion regulation process serving as an internal resource, such as inner hearing of any song associated with positive memories or associations. For explicit regulation, music involves a conscious effort to evoke changes in emotional valence, such as listening to uplifting music for affective entrainment. Deliberately selecting music for listening or playing is a common example of using music as an explicit emotion regulation strategy. Bogert et al. [17] demonstrated that different areas of the brain are activated depending on what the listener focuses on during music listening. When they attend to the actual instrumentation of the music piece (implicit processing), regions involved in emotional processing are activated. Conversely, when they concentrate on the emotional content of the music (explicit processing), regions specifically governing emotional processing are activated.

The regulatory process can be approached through either a bottom-up or top-down method, depending on the musical elements employed. In the bottom-up approach, rhythm can be utilized to entrain physiological states, thereby uplifting mood [18,19]. Conversely, in the top-down approach, lyrics or predictable musical progression in the melody can help relax and alleviate distressing states [20,21].

Research supports that rhythm as one of the profound components of music governs arousal level, manifested by the listener’s physiological variable. One of the main principles that support music’s regulatory effect on emotion may be entrainment. Music can entrain physiological states through its rhythmic characteristics [22]. Studies also show that valence and arousal are two constructs that affect each other as well. Further, the combined form of musical components may have the potential to evoke a holistic effect in generating emotions. However, there is a notable scarcity of research on how these musical elements interact synergistically to induce emotional responses.

In the last decades, the field of music emotion regulation has emerged as a distinctive area of study, emphasizing the role of music in regulating mood, enhancing positive emotional states, and reducing negative emotions [15]. In contrast to more established emotion regulation (ER) fields, the field of music emotion regulation (MER) lacks a unified and agreed-upon theoretical framework for examining how music regulates emotions [23].

This study sought to explore the realm of knowledge concerning the regulatory effects of music on emotions, focusing on the use of music to induce emotion regulation in the existing studies on music emotion regulation. To achieve this, a scoping review method was employed. Unlike systematic reviews, which aim to synthesize results for end-users, scoping reviews cover a broader body of literature to clarify evidence in certain areas of knowledge [24]. As music emotion regulation (MER) is becoming a field with practical implications, this scoping review aims to identify how music and its intra-musical constructs are utilized for regulatory effects on human emotion across different literature.

This study aimed to examine the concept of the regulatory effect of music on emotion, including how emotion is defined and what is the rationale behind music selection purported to induce the regulatory effect. The study observed the following five parts pertaining to music emotion regulation studies:

1 Research trend in MER studies: This part identifies the prevailing trends and patterns in MER studies over time.
2 Scoping emotion in MER studies: This part explores how emotions are conceptualized and operationalized within the context of MER studies, including specific definitions related to emotional responses to music.
3 Scoping music and its components in MER studies: This part examines the rationale and working mechanisms behind selected music and its components.
4 Scoping the regulatory process of emotion in MER studies and domains: This part compares the definitions of regulation between emotion regulation (ER) literature and MER studies and suggests the domains where these regulatory processes occur.
5 Scoping challenges for components of MER studies: This part summarizes key findings and challenges identified from the scoping review regarding the three components of MER studies, and highlights areas for future research.

## 2. Method

To examine a comprehensive concept of music emotion regulation, the researchers adopted a scoping review methodology. A scoping review, as defined by Arskey and O’Malley [25], aims to map the key concepts that underpin a specific research area. The study purported to explore various concepts and methodological sources that exist in music emotion regulation studies, identifying trends, gaps, and challenges for further investigation.

### 2.1. Step 1: Studies Included for the Review

The scoping review methodology is designed to be inclusive and informative, providing a broad overview of the existing research on the subject. By following these steps, the authors aimed to conduct a comprehensive analysis of the literature related to music, emotion, and regulation. Accordingly, to identify which studies to include, the authors established inclusion and exclusion criteria based on guidelines suggested by the JBI Manual for Evidence Synthesis [26] (Table 1):

1. Keywords: The authors used three main keywords for their search—“music”, “emotion”, and “regulation”—in various combinations using Boolean operations AND to identify relevant literature. These keywords helped narrow the search to articles specifically related to use of music for regulatory effect on music. By using “regulation” as a keyword, the authors distinguished studies focused on music emotion regulation (MER) from broader music emotion (ME) studies, which generally explore a wide range of emotional responses to music.
2. Electronic databases: The authors conducted a search through various electronic databases, including PubMed, PsychINFO, Web of Science, and Scopus. These databases are reputable sources for academic research articles across various fields.
3. Scope of Search: The search was limited to peer-reviewed academic journals published in the English language. This restriction ensures the credibility and quality of the sources.
4. Timeframe: The search covered articles published from 2014 to 2024. This range was chosen to include recent research while avoiding outdated studies.
5. Manual selection: To ensure that all relevant articles were included, the authors manually searched representative journals in the field. These journals include *Musicae Scientiae*, *Psychology of Music*, *Cognition & Emotion*, *Journal of Music Therapy*, and *Nordic Journal of Music Therapy*. This step was crucial for identifying articles that may not be available in the electronic databases.

### 2.2. Step 2: Study Selection

**Figure 1:**
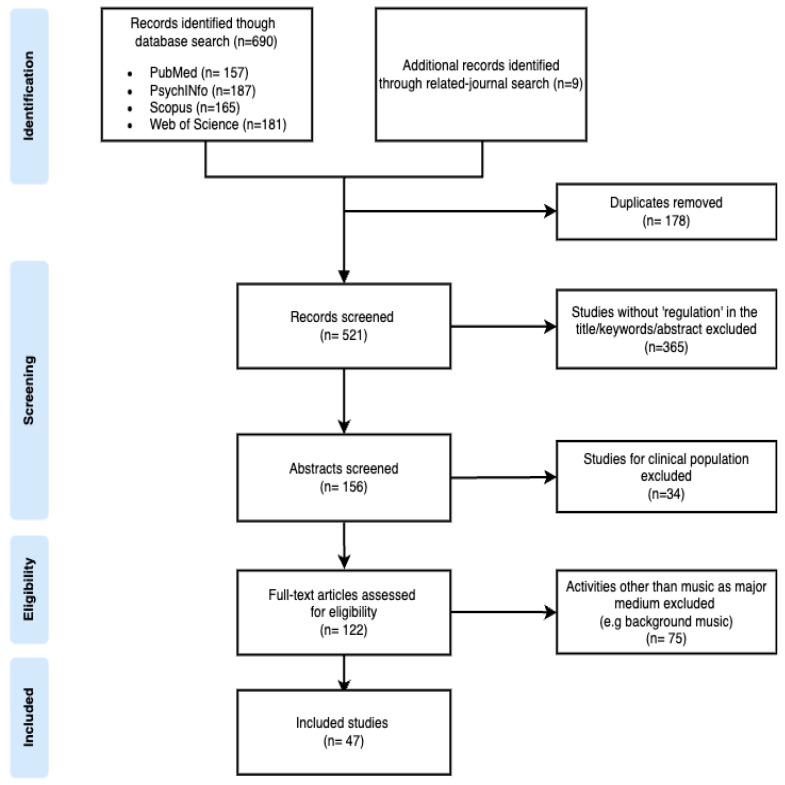
Flow diagram of selecting included studies.

After conducting a search, the authors independently screened studies retrieved to determine whether they met the selection criteria for eligibility. Studies with insufficient information were examined through a full-text review to confirm their inclusion. Three authors evaluated the studies independently according to the predetermined inclusion and exclusion criteria. Subsequently, the authors collaboratively reviewed the studies until a consensus on inclusion was achieved. The flow chart for the article selection process is provided below. Each step sorted the studies, and ultimately, 47 studies were selected (Figure 1).

### 2.3. Step 3: Charting the Data

Based on the exclusion and inclusion criteria, a total of 47 articles were selected for the scoping review. Each article was reviewed and categorized according to specific criteria, including the year of publication, participants (e.g., age, demographics), study design (e.g., experimental, observational), and other relevant characteristics including the following:

1. Music selection and implementation: The authors analyzed the selection criteria of music and rationale for formulating music activities described in each article. This analysis includes identifying the types of music activities used in the studies, such as listening to music, playing musical instruments, singing, or a combination of these activities. They also noted specific details about the musical pieces used for emotion regulation, including duration of music played, number of pieces used, and whether the music was selected by the researcher or the participants.
2. Regulation: The authors analyzed the operational definitions of regulation and how the studies intended to measure music’s regulatory effect. The authors further investigated variables that manifested the changes in the emotion as a regulatory effect.
3. Emotion: The review examined how emotion was defined as a dependent variable. Additionally, other synonyms used interchangeably with “emotion” were examined (Appendix B).

### 2.4. Step 4: Summarizing and Reporting Results

Initially, three authors independently conducted data extraction from the included studies. Subsequently, they iteratively refined and reviewed the identified categories through multiple discussions to ascertain the final agreement to derive the results.

Following a thorough analysis of studies and identifying the scoping theme, the authors used the Preferred Reporting Items for Systematic Reviews and Meta-Analyses extension for Scoping Reviews (PRISMA-ScR) checklist to ensure adherence to the steps and categories of the scoping theme [27]. The PRISMA-ScR checklist provides a recommended guideline for reporting the findings of scoping reviews.

## 3. Results

The study purported to examine the use of music for emotion regulation, as explored in various studies to construct an understanding of music’s regulatory effect on emotion. A scoping review is used in order to identify the knowledge gap, identify concepts, and investigate scoping challenges in conducting research on music emotion regulation (MER) studies. The findings of reviews are presented for each research question.

### 3.1. RQ1: Trends of Music Emotion Regulation Studies

The general characteristics of the 47 selected publications are as follows (Table 2). The trend of studies in music emotion regulation has increased gradually over time. From 2014 to 2016 and between 2017 and 2020, nine studies (19.1%) were published in each period. Most recently, from 2021 to 2024, 29 (61.8%) articles were published, and this significant increase, including the results from the first half of 2024, indicates that research in the field of music emotion regulation is currently very active.

The review showed that the primary music activity for emotion regulation was listening, which accounted for 40 out of 47 studies (85.1%). Following listening, playing the instruments and a combination of various music activities were each implemented in only three studies, representing 6.4% total. Singing was reported in one study.

Regarding the relationship between sample size and experiment setting, studies conducted in experimental environments with between 30 and 99 participants had the highest proportion, accounting for 15 studies (31.9%). On the other side, among the studies that examined emotion regulation through the use of music in everyday life, the largest number of studies (23.4%) involved between 300 and 999 people. In addition, studies often utilized larger sample sizes in both experimental and natural environments. Notably, studies involving more than 1000 participants typically used a specific mobile application to engage with music in everyday life (Table 3). This indicates that while some studies employed experimental designs, a significant portion was conducted in real-world, daily settings.

A total of 47 studies utilized self-report and physiological methods to measure changes in emotion regulation. Forty (85.1%) studies utilized self-report methods, two (4.2%) studies employed physiological measurement methods, and five (10.7%) studies used a combination of self-report and physiological measurement.

Among the studies that used self-report methods, questionnaires and standardized scales were the most common tools. For experience sampling methods (ESMs), a tool to collect real-time data on daily musical experience was used in four studies, and interviews were conducted for qualitative data. For physiological methods, EEG, fMRI, ECG, SCL, EMG, the Face–Word Stroop Task, EDA, and saliva cortisol levels were used for measurement (Table 4).

### 3.2. RQ2: Scoping Emotion in MER Studies

In the 47 selected studies, emotion was typically treated as a dependent variable encompassing a wide spectrum, ranging from somatic responses to behavioral reactions. The term “emotion” was frequently used interchangeably with synonyms or alternative expressions such as mood or affective state and stress response, and they were consistent with terms outlined in [7].

The most frequently used terms were “emotional response or reaction”, accounting for 24 out of 47 studies (51%). “Stress responses or stressful feeling” was used in 11 studies (23.4%), where researchers tried to examine the effect of music on emotion regulation especially when the participants were in a negative situation. “Affects or affective states” and “mood or mood state” were each used in six studies (12.8%) (Table 5).

### 3.3. RQ3: Scoping Music and Its Components in MER Studies

Regarding the selection criteria of music and music engagement time, only 15 (32%) of the reviewed studies mentioned the number of music pieces used or the time of the music engagement. For the rationale/reasons for the selected music activity, 38 (80.9%) studies specified the reasons, referencing existing emotion studies rather than presenting a distinct rationale specific to their own research. On the other hand, the remaining nine studies did not mention any reasons for music activities at all (Table 6).

These findings suggest that there is a lack of a theoretical rationale for musical components and their regulatory effect on emotion. Music for any form of implementation or intervention is particularly difficult to fully describe due to the complexity of music stimuli (e.g., rhythm, pitch, tempo, harmonic structure, timbre). Additionally, it is also challenging to specifically elaborate on the music experiences (e.g., active music making vs. receptive music listening) and other factors unique to its experiential aspects. Consequently, many studies treated music as a single “entity variable”, often neglecting to verify the intra-musical variables involved in their interventions.

Secondly, the duration of music implementation varied tremendously across studies without any specific rationale, allowing listeners subjective control over time, conditions, and the environment, as well as the use of devices (live, recorded, headphones, speakers, etc.). Researchers may have intended to observe the music’s regulatory effect in naturalistic, real-life settings. However, using participants’ personal music pools may weaken the scientific foundation for assessing music’s regulatory effect on emotion, as musical selections vary widely. When the music selection lies in one’s open and subjective music preferences, the study naturally shifts its focus toward examining the regulatory effect from a listener-related orientation rather than a music-related one. In such cases, it becomes crucial to include information not only about the individual’s selected music but also about their motives for choosing and engaging with the music, as these factors contribute to their awareness of any emotional changes. Moreover, a naturalistic environment can also yield confounding variables in the listening condition, such as music being played in the background rather than as a primary focus. This factor can complicate the interpretation of how music directly affects emotional regulation.

### 3.4. RQ4: Defining Regulatory Process of Emotion

The review also examined the operational definitions of regulation used in the context of music emotion regulation (MER), comparing them with those found in the literature on emotion regulation (ER). While most of the terms suggested by the ER literature were adopted by MER studies, demonstrating consistency in the strategic concepts, there were unique terms specific to MER, implying music’s inherent effect. Four clusters of wordings that described the direction of regulation were derived. The first cluster had the most common terms used in MER studies, such as “change”, “modify”, “improve”, “enhance”, “modulate”, “replace”, “alter”, and “adjust”. The second cluster of terms in MER studies was “control”, “manage”, “maintain”, “sustain”, “focus”, and “down-regulate”, implying strategies aimed at holding or containing emotions. The third cluster of terms included expressions like “diversion”, “distraction”, and “expression”, which indicate shifting attention toward alternatives or substitutes. This approach contrasts with strategies that involve containing or holding emotions.

Lastly, the fourth cluster included terms related to the nature of “discharge”, “venting”, “releasing”, and “eliminating” negative affect, connoting a concept of cleansing difficult emotions. Interestingly, these terms differ from those commonly found in the existing literature on emotion regulation that does not involve music. This finding suggests that music possesses a unique mechanism facilitating the release of difficult emotions, thereby leading to a cathartic form of regulation. In other words, music may enable a distinctive process of emotional cleansing, offering a means to alleviate and manage negative emotions in a way that is not typically addressed by other regulatory strategies (Table 7).

The review further examined where and how the regulatory process occurred during or after music engagement. A consensus emerged across many studies that this process is multifaceted, spanning various domains from initial sensation to triggered expressive action or behavior. Specific processes associated with each domain are delineated in Table 7. These domains include physiological, psychological, affective, cognitive, and behavioral aspects. What these findings reveal is the multi-leveled nature of music’s regulatory process, which evolves across various domains, often simultaneously, highlighting the interconnectedness of these dimensions [36].

The findings of the review showed that at the physiological level, music is involved in down-regulating negative emotions, heightened arousal, or uplifting energy levels. At the psychological level, anxiety and stress responses are key variables that are subject to modulation or sedation. In the affective domain, changes in emotions are involved, whether suppressed, induced, or expressed, including coping strategies to improve or stabilize mood. At the cognitive level, the focus is on mental processes such as conscious effort, reappraisal, rumination, or introspection. Webb, Miles, and Sheeran [11] identified this mechanism as particularly effective among emotion regulation strategies. Lastly, the behavioral domain involves activated behaviors such as vocal or physical expression that manifests intense reactions to music. This expressive behavior may involve ventilation or the cathartic discharge of emotions, ultimately leading to a renewed sense of revival [46,81]. This is consistent with the findings of studies that suggest ER is a multi-level process that involves a whole-body emotion system, including subjective feelings, cognition, physiological and neural systems, and behavioral responses [36,37,82] (Table 8).

### 3.5. RQ5: Scoping Challenges for MER Studies

The scoping review findings showed categories of challenges pertaining to the three components of music, emotion, and regulation. Firstly, music as the major medium for regulatory effect, including its intra-musical components, should be thoroughly discussed in the context of music selection criteria. One of the recent fidelity analyses of MER studies showed that the replicability of the experiment was weak due to insufficient information on the music rationale. In order to substantiate well-grounded statements on the logical mechanism of music’s regulatory effect on emotion, enhancing the fidelity and rationale of music’s usage in the MER studies is crucial [23,83]. This challenge includes providing comprehensive details concerning music implementation and data collection methods to study regulatory changes [84,85].

Secondly, given that the primary objective of MER is to bring emotional change, there is a need for a more robust theoretical definition of the term “emotion”. This definition should encompass how emotions are assessed and measured to capture the changes that occur. Furthermore, it is crucial for studies to clarify whether participants are reporting on the emotions they “felt” or those they cognitively appraised or “perceived” in music, as these represent two distinct dimensions (somatic vs. cognitive) of identifying emotions.

Lastly, there must be a clear mechanism linking the selected music intervention to emotion regulation. Despite the studies aiming to induce emotion regulation through music, there was insufficient information regarding the mechanism by which music exerts its regulatory influence. Measuring the regulatory effect presents significant challenges in both ER and MER studies [11]. However, when MER studies specifically target the regulatory impact of music, the procedure must be meticulously designed (Table 9).

## 4. Conclusions

Music emotion regulation (MER) is emerging as a distinct research area that focuses on music as a deliberate and functional tool for modulating and regulating emotions. The scoping review of current studies aimed to explore the use of music for emotion regulation. Music inherently serves as a temporal art of emotion, and it acts as a catalyst and a regulatory medium, augmenting strategies for facilitating emotion regulation. The results indicate minimal distinction between the concepts of music emotion (ME) and music emotion regulation (MER) studies.

Also, deeper contemplation of intra-musical elements that constitute music would be imperative to build a robust theoretical foundation for music’s regulatory mechanism. This would lead to establishing a more compelling mechanism behind music’s “regulatory effect” in order to firmly establish the concept of music emotion regulation (MER) as a unique research area.

The limitation of this study lies in locating all existing literature on music emotion regulation and determining which articles align with the scope of this study. Each article had to undergo a careful review individually for inclusion, even if it contained the target keywords in its title. Often the authors may have had different perceptions regarding the terms associated with them, and therefore, some studies turned out to be irrelevant after the review.

Conclusively, the findings from this study offer exploratory insights and point toward the need for further refinement of the concept of MER for its practical application. While the study provides valuable directionality, it is essential to acknowledge that the field of MER is evolving and being refined, especially the regulatory mechanism. Future research may uncover additional knowledge that was not captured in this initial review.

## Figures and Tables

**Table 1:** Inclusion and exclusion criteria for the scoping review.

Item	Inclusion Criteria	Exclusion Criteria
Subjects	Participants: Non-clinical subjects.	Studies for clinical subjects.
Scope of Keywords	Music: Studies employing various music activities, such as listening, singing, playing, or combined as a primary intervention.	Studies using activities other than music. Studies that used music as an indirect or secondary medium such as meditation, progressive relaxation, etc.
	Regulation: Studies that purported to examine the regulatory effect of music.	Studies on music and emotion in general, without clear purpose of “regulation”. Studies on general emotional response to music.
	Emotion: Studies that aimed to bring changes in emotional realm, including other synonyms such as feeling, mood, affect, etc.	Studies that examined general responses inclusive of emotion, such as quality of life, behavioral management, etc.
Source Types	Studies published in English with quantitative, qualitative, and mixed methodologies.	Studies on the efficacy of music on emotion, such as meta-analysis.

**Table 2:** Publication year and music activities of studies (*N* = 47).

Factors	Characteristic	No. (%)	Studies
Publication Year	2014–2016	9 (19.1)	[28,29,30,31,32,33,34,35,36]
	2017–2020	9 (19.1)	[37,38,39,40,41,42,43,44,45]
	2021–2024	29 (61.8)	[46,47,48,49,50,51,52,53,54,55,56,57,58,59,60,61,62,63,64,65,66,67,68,69,70,71,72,73,74]
Music Activities	Listening	40 (85.1)	[28,30,31,32,33,34,35,36,37,38,39,40,41,43,44,45,46,47,48,49,50,52,53,55,56,57,58,59,60,63,64,65,66,68,69,70,71,72,73,74]
	Singing	1 (2.1)	[42]
	Playing	3 (6.4)	[61,62,67]
	Combined	3 (6.4)	[29,51,54]

**Table 3:** Conditions for music engagement: experimental vs. non-experimental condition (*N* = 47).

Music Engagement	Sample Size	No. (%)	Studies
Experiment	Less than 30	2 (4.3)	[58,60]
	30–99	15 (31.9)	[32,36,37,39,41,43,44,47,49,50,59,63,67,71,72]
	100–299	4 (8.5)	[28,45,73,74]
	300–999	1 (2.1)	[57]
	Over 1000	1 (2.1)	[42]
Non-experimental (Daily, etc.)	Less than 30	3 (6.3)	[52,64,70]
	30–99	2 (4.3)	[30,31]
	100–299	6 (12.8)	[34,35,38,46,55,69]
	300–999	11 (23.4)	[29,33,40,48,51,53,56,61,62,66,68]
	Over 1000	2 (4.3)	[54,65]

**Table 4:** Methods for measuring emotion regulation (*N* = 47).

Measurement		Studies
Self-report (n = 40)	Questionnaire	[28,29,30,31,32,33,34,35,36,37,38,39,40,41,42,43,45,46,48,49,50,51,52,53,54,55,56,57,59,61,62,63,64,65,66,67,68,69,70,71,72,73,74]
	Experience sampling method	[33,64,69,70]
	Interview	[44,60]
Physiological measurement (n = 2)	EEG, fMRI	[28,47,58,59]
	ECG	[36]
	SCL, EMG	[36,37]
	The Face–Word Stroop Task	[59]
	EDA	[37]
	Saliva cortisol	[71]
Combined (n = 5)		[28,36,37,59,71]

EEG: electroencephalograms, fMRI: functional magnetic resonance imaging, ECG: electrocardiography, SCL: skin conductance levels, EMG: facial electromyography, EDA: electrodermal activity.

**Table 5:** Terms used to define emotion (*N =* 47).

	Keywords	No. (%)	Sample Studies
Emotion	Emotional response or reaction	24 (51)	[30,31,32,33,35,36,39,40,41,42,44,45,46,49,57,58,60,61,62,64,67,71,72,74]
	Mood or mood state	6 (12.8)	[34,50,52,55,70,73]
	Affects or affective states	6 (12.8)	[28,29,38,47,56,69]
	Stress response or stressful feeling	11 (23.4)	[37,43,48,51,53,54,59,63,65,66,68]

**Table 6:** Information regarding music engagement in MER studies (*N* = 47).

	Characteristics	No. (%)	Sample Studies
Music Engagement Time	Specified	25 (53.2)	[28,32,33,34,36,37,41,43,44,45,47,50,53,57,58,59,62,66,67,69,70,71,72,73,74]
	Not Specified	22 (46.8)	[29,30,31,35,38,39,40,42,46,48,49,51,52,54,55,56,60,61,63,64,65,68]
Number of Music Pieces	Specified	22 (46.8)	[28,31,32,36,37,39,40,43,44,45,47,49,58,59,60,61,62,70,71,73,74]
	Not Specified	25 (53.2)	[29,33,34,35,38,41,42,46,48,50,51,52,53,54,55,56,57,63,64,65,66,67,68,69,72]
Music Engagement Time and Number of Music Pieces	Both Specified	15 (32)	[28,32,36,37,43,44,45,47,58,59,62,70,71,73,74]
	Both Not specified	16 (34)	[29,35,38,40,42,46,48,51,52,54,55,56,63,64,65,68]
	One only	16 (34)	[30,31,33,34,39,41,49,50,53,57,60,61,66,67,69,72]
Reasons for Selected Music Activities	Specified	38 (80.9)	[29,30,31,33,34,35,36,37,38,41,42,43,44,45,46,49,50,52,53,55,56,57,59,60,61,62,63,64,65,66,67,68,69,70,71,72,73,74]
	Not Specified	9 (19.1)	[28,32,39,40,47,48,51,54,58]

**Table 7:** Key terms defining music’s regulatory function in relation to definitions of emotion regulation (*N* = 47).

Term		Keywords of Music Emotion Regulation (MER) Studies Adopted	Studies
Terms for direction of regulation	Cluster 1: Changing and Modifying Titrate, alter, modulate response [7,75] Coping [76]	Change/modify/improve/enhance/modulate/replace/alter/adjust	[28,33,37,39,41,44,46,48,52,55,64,65,67,70,71]
		Increase arousal/maximize	[37,40,62]
		Modulation of thoughts/affect/behavior	[28,38,61]
		Coping	[53,61]
		Induce/influence	[38,39,51,60]
	Cluster 2: Holding and Maintaining Management of generated emotion [77] Track, assess, control [67] Dampen, maintain, intensify [6]	Control/manage	[49,65,67]
		Maintain/sustain	[28,29,37,46,54,55,64]
		Focus	[14,40,46]
		Reduce/diminish/down-regulate/decrease/minimize	[36,37,52,56,62,68,70]
	Cluster 3: Distracting and Diverging Distraction [78] Diversion [79] Reroute [67] Emotion Expression [80]	Diversion/distracting	[45,56]
		Expression	[39,55,60,62]
	Cluster 4: Discharging and Venting	Discharge/venting/releasing/eliminating	[37,54,56,65]

**Table 8:** Domains involved in emotion regulation process using music (*N* = 47).

Domain	Regulatory Processes	Sample Studies
Physiological	Down-regulate, heightened arousal, energy	[28,31,36,37,39,40,41,43,47]
Psychological	Modulation of anxiety and stress	[42,48,51,63,68]
Affective	Changes in the generated emotion Improve, control, and maintain mood	[31,32,34,35,36,38,39,41,45,46,47,51,52,53,55,56,57,59,60,66,69,70,71,72,73,74]
Cognitive	Mental process that involves conscious effort, reappraisal, rumination, or introspection	[29,30,31,33,49,52,53,57,58,59,63,67,68,74]
Behavioral	Expressive action or any behavioral expression Intense reactions to goal-oriented changes	[28,31,32,37,48,59,60,61,64]

**Table 9:** Scoping challenges for each component for further MER studies.

Components	Agendas	Scoping Challenges and Considerations
Emotion	Working definition	Define in relation to other synonyms: feeling, emotion, mood, affect, etc.
	Measurement	Changes in the emotion: perceived (cognitive appraisal) vs. felt (embodied)
	Components/variables	Suggest constituting concepts: valence, arousal, intensity, etc.
Music	Musical elements	Rationale for intra-musical elements: tempo/rhythmic idiom/tonal idiom/other components
	Exposure time and engagement manner	Number of excerpts and duration of each piece
		Specify listening time, condition, situation, etc.
	Selection rationale	Specify researcher’s stance pertaining to music’s facilitative strategy for emotion regulation
Regulation	Regulatory mechanism of music	Provide working rationale and mechanism linking music intervention and emotion

## Data Availability

No new data were created or analyzed in this study. Data sharing was not applicable in this study.

## Appendix A. List of Included Studies

	**Author (Year)**	**Title**	**Keywords**
[28]	Carlson et al. (2015)	Maladaptive and Adaptive Emotion Regulation Through Music: A Behavioral and neuroimaging Study of Males and Females	Music, emotion regulation, fMRI, prefrontal cortex, gender differences, mental health
[29]	Chin and Rickard (2014)	Emotion Regulation Strategy Mediates Both Positive and Negative Relationships Between Music Uses and Well-being	Emotion regulation, reappraisal, suppression, music engagement, well-being
[30]	Dingle et al. (2016)	Tuned In Emotion Regulation Program Using Music Listening: Effectiveness for Adolescents in Educational Settings	Emotion regulation, emotion awareness, music, engagement, adolescents
[31]	Fernández-Sotos et al. (2016)	Influence of Tempo and Rhythmic Unit in Musical Emotion Regulation	Emotion regulation, music note value, tempo, rhythmic unit
[32]	Medrano et al. (2016)	Effects of Induction of Positive and Negative Emotional States on Academic Self-Efficacy Beliefs in College Students	Emotion, self-efficacy, emotion regulation
[33]	Randall et al. (2014)	Emotional outcomes of regulation strategies used during personal music listening	Emotion regulation, experience sampling method, hedonic shift, mental health, mobile phone, music, well-being
[34]	Shifriss et al. (2015)	When you’re down and troubled: Views on the regulatory power of music	Happy music, mood-matching music, mood regulation, music preferences, music therapy, positivity effect, sad music
[35]	Van den Tol et al. (2016)	Sad Music as a Means for Acceptance-Based Coping	Acceptance, aversive situations, coping, emotion, sad music, sadness, self-regulation
[36]	White and Rickard (2016)	Emotion Response and Regulation to “Happy” and “Sad” Music Stimuli: Partial Synchronization of Subjective and Physiological Responses	Affect regulation, conductance, emotional response, heart rate, multicomponent, skin, subjective, synchronized
[37]	Baltazar et al. (2019)	Is It Me or The Music? Stress Reduction and The Role of Regulation Strategies and Music	Affect regulation, emotion regulation, music, musical mechanisms, relaxation, self-chosen music, strategies, stress, tension
[38]	Chang et al. (2020)	Music major, affects, and positive music listening experience	Music major, emotion regulation, positive affect, negative affect, music listening, contemplation
[39]	Cheng (2020)	Empirical Analysis on The Influence of Music Speed and Music Mode on The Emotions of College Students	Music speed, music mode, college students, emotional regulation
[40]	Cook et al. (2019)	Music as an Emotion Regulation Strategy: An Examination of Genres of Music and Their Roles in Emotion Regulation	Affect, emotion regulation, emotions, music
[41]	Dingle and Fay (2017)	Tuned in: The effectiveness for young adults of a group emotion regulation program using music listening	Emotion regulation, engagement, music, young adults
[42]	Fancourt and Steptoe (2019)	Present in Body or Just in Mind: Differences in Social Presence and Emotion Regulation in Live vs. Virtual Singing Experiences	Music, emotion regulation, emotions, technology, social
[43]	Groarke et al. (2020)	Does Listening to Music Regulate Negative Affect in a Stressful Situation? Examining the Effects of Self-Selected and Researcher-Selected Music Using Both Silent and Active Controls	Anxiety, coping, mindfulness, music listening, regulation, stress
[44]	Saarikallio et al. (2017)	Adolescents’ musical relaxation: Understanding related affective processing	Relaxation, music listening, adolescents, affect, emotion regulation strategies, emotion induction mechanisms
[45]	Shifriss et al. (2020)	Don’t let me down: The effect of age and chosen music on mood is moderated by focus on emotions	Age, attention deployment, emotion regulation, positive affect (PA), sad music
[46]	Bachman et al. (2022)	Emotion Regulation Through Music and Mindfulness are Associated with Positive Solitude Differently at The Second Half of Life	Emotion regulation through music listening, PS, loneliness, mindfulness, second half of life
[47]	Berthold-Losleben et al. (2021)	A short-term Musical Training Affects Implicit Emotion Regulation Only in Behaviour But not in Brain Activity	Music, olfaction, FMRI, emotion regulation, training, affective rivalry, multisensory integration, listening, implicit
[48]	Carlson et al. (2021)	The Role of Music in Everyday Life During the First Wave of the Coronavirus Pandemic: A Mixed-Methods Exploratory Study	Music, coronavirus, music listening, anxiety, emotion regulation
[49]	Carvalho et al. (2022)	The “Ifs” and “Hows” of the Role of Music on the Implementation of Emotional Regulation Strategies	Emotional regulation, music listening, musical sophistication, executive functions, empathy
[50]	Coulthard et al. (2023)	Music as an Alternative Self-regulation Strategy to Snack Foods Following a Negative Mood Induction in 5–7-year-old Children: Interactions with Parental Use of Food as a Reward	Music, emotional eating, children, emotional regulation, reward
[51]	Ferreri et al. (2021)	Engagement in Music-Related Activities During the COVID-19 Pandemic as a Mirror of Individual Differences in Musical Reward and Coping Strategies	COVID-19, music reward, emotional regulation, musical abilities, individual differences
[52]	Garrido et al. (2022)	Music Listening and Emotion Regulation: Young people’s Perspectives on Strategies, Outcomes and Intervening Factors	Music, emotion regulation, depression, anxiety
[53]	Gibbs and Egermann (2021)	Music-Evoked Nostalgia and Wellbeing During the United Kingdom COVID-19 Pandemic: Content, Subjective Effects, and Function	Nostalgia, COVID-19, listening, music, wellbeing, emotion, regulation, lockdown
[54]	Granot et al. (2021)	“Help! I Need Somebody”: Music as a Global Resource for Obtaining Wellbeing Goals in Times of Crisis	Music, COVID-19, wellbeing, individualistic and collectivistic cultures, mood regulation, nostalgia, gender, age
[55]	Jakupčević et al. (2021)	Music As a Tool for Mood Regulation: The Role of Absorption vs. Mindfulness	Absorption in music, mood regulation, mindfulness, musical taste
[56]	Koehler et al. (2023)	The Interplay Between Music Engagement and Affect: A Random-Intercept Cross-Lagged Panel Analysis	Music listening, music making, mood regulation, emotions
[57]	Larwood and Dingle (2022)	The Effects of Emotionally Congruent Sad Music Listening in Young Adults High in Rumination	Music, emotion regulation, rumination, musical emotion mechanisms
[58]	Li and Zheng (2021)	Emotion Recognition and Regulation Based on Stacked Sparse Auto-Encoder Network and Personalized Reconfigurable Music	EEG, reconfigurable music, personalized emotion regulation, SSAE, personalized music-EEG library
[59]	Liu et al. (2021)	Regulation of Mindfulness-Based Music Listening on Negative Emotions Related to COVID-19: An ERP Study	Mindfulness meditation, music listening, emotion regulation, cognitive control, ERPs
[60]	Loureiro et al. (2024)	Why I Listen to Music: Emotion Regulation and Identity Construction Through Music in Mid-adolescence	Music, mid-adolescence, identity development, emotion regulation, narratives
[61]	Madden et al. (2023)	Emotional Cherry Picking: The Role of Personality and Goal Orientation in Selective Emotion Regulation for Musical Practice	Emotion regulation, musical practice, personality, trait-dependent, mastery goal
[62]	Madden and Jabusch (2021)	Instrumental and Hedonic Motives for Emotion Regulation in Musical Practice	Musical practice, emotion regulation, meta-emotion beliefs, goal orientation
[63]	Maidhof et al. (2023)	Effects of Participant-Selected Versus Researcher-Selected Music on Stress and Mood-The role of gender	Stress, mood, participant-selected music, researcher-selected music, emotions, emotion regulation strategies
[64]	Ma’rof et al. (2023)	Gender Differences in The Function of Music for Emotion Regulation Development in Everyday Life: An Experience Sampling Method Study	Emotion regulation, gender differences, music listening, strategies and mechanisms
[65]	Martín et al. (2021)	Music As a Factor Associated with Emotional Self-Regulation: A Study on Its Relationship to Age During COVID-19 Lockdown in Spain	Music, COVID-19, pandemic, emotional self-regulation, age
[66]	Martínez-Castilla et al. (2021)	The Efficacy of Music for Emotional Wellbeing During The COVID-19 Lockdown in Spain: An Analysis of Personal and Context—Related Variables	COVID-19, music, efficacy, emotional wellbeing, affect regulation, Spain
[67]	Nwokenna et al. (2022)	Effect of Educational Music Intervention on Emotion Regulation Skills of First-year University Music Education Students	Educational music intervention, emotion regulation, emotion regulation skills, first-year music education students, rational humorous song
[68]	Park and Suh (2023)	Hardiness and Expectations for Future Life: The Roles of Perceived Stress, Music Listening for Negative Emotion Regulation, and Life Satisfaction	Hardiness, stress, music listening, life satisfaction, expectations for future life
[69]	Randall et al. (2022)	Success in reaching affect self-regulation goals through everyday music listening	Music and emotion, emotion regulation, experience sampling method, everyday music listening
[70]	Taruffi (2021)	Mind-Wandering during Personal Music Listening in Everyday Life: Music-Evoked Emotions Predict Thought Valence	Mind-wandering, music-evoked emotions, visual mental imagery, mood regulation, health, wellbeing, digital health interventions, experience sampling method, personal music listening
[71]	Tervaniemi et al. (2021)	Psychological and Physiological Signatures of Music Listening in Different Listening Environments—An Exploratory Study	Music, emotion, emotion regulation, stress reduction, cortisol
[72]	Vidas et al. (2023)	Efficacy of the Tuned In music emotion regulation program in international university students	International university students, emotion regulation, music listening, intervention, well-being
[73]	Völker (2021)	Personalising Music for More Effective Mood Induction: Exploring Activation, Underlying, Mechanisms, Emotional Intelligence, and Motives in Mood Regulation	BRECVEMA framework, emotional intelligence (EI), music in mood regulation (MMR), musical mood induction procedure (MMIP), reciprocal-feedback model (RFM)
[74]	Völker (2022)	Measuring Emotional Music Experience: Spreading Activation and BRECVEMA Mechanisms	Music perception, spreading activation, BRECVEMA mechanisms, empathizing-systemizing, emotion regulation

## Appendix B

**Author (Year)**	**Country**	**Study Design**	**Participant**			**Type of Music Activity**			**Duration of Music Activity**	**Partipication Conditions of Music Activites**	**Aims of the Study**	**Measurement**	**Outcome**
			**N**	**Age**	**Characteristics**	**Listening**	**Singing**	**Playing**					
Carlson et al. (2015) [28]	Finland	Quant	123	18–55	Participants from the Helsinki area recruited through emails and flyers	o			4 s	Experiment	To explore the relationship between self-directed music listening strategies and mental health, specifically examining the effects of these strategies on depression, anxiety, and neuroticism, and their neural correlates using fMRI.	Self-report (Questionnaire), fMRI	Discharge Strategy: Anxiety and neuroticism increased by using music to express negative emotions, particularly in males. Diversion Strategy: mPFC activity in females increased by using music to distract from negative emotions.
Chin & Rickard (2014) [29]	Australia	Quant	637	20–58	Recruited via posters placed in several music venues and tertiary institutions providing music courses in Melbourne, Australia.	o	o	o	NS	Everyday	To examine the mediating effects of emotion regulation strategies (reappraisal and suppression) on the relationship between music engagement and well-being.	Self-report (Questionnaire)	The path of mediation was dependent on the type of emotion regulation strategy utilized, as well as the way in which one engages with music.
Dingle et al. (2016) [30]	Australia	Quant	41	14–17	Member of the BoysTown experiential learning program in a regional city in Australia	o			NS	Everyday	To evaluate the effectiveness of “Tuned In,” a novel emotion regulation intervention using participant-selected music to evoke emotions and teach emotional awareness and regulation skills in adolescents.	Self-report (Questionnaire)	Pre- to post-program improvements in emotion awareness, identification, and regulation among both “at risk” adolescents and mainstream secondary school students.
Fernández-Sotos et al. (2016) [31]	Spain	Quant	63	19–29	Young people	o			NS	Everyday	To investigate how musical cues related to note value, specifically tempo and rhythmic unit, influence the regulation of emotional states in listeners.	Self-report (Questionnaire)	Increasing tempo from 90 to 150 bpm significantly enhanced feelings of happiness, surprise, tension, expressiveness, and amusement while reducing sadness. Using sixteenth notes produced the highest emotional impact in terms of these emotions, whereas whole and half notes resulted in lower values.
Medrano et al. (2016) [32]	Argentina	Quant	50	17–31	College students	o			10 min	Experiment	To examine the relationship between positive and negative emotions and self-efficacy, considering participants’ difficulties in emotional regulation as a co-variable.	Self-report (Questionnaire)	Inducing positive moods increases self-efficacy, while negative moods decrease it. This was seen in participants with intense moods and those with a typical character items.
Randall et al. (2014) [33]	Australia	Quant	327	*M* = 21.02	Participants recruited through MuPsych smartphone app	o			2 weeks	Everyday	To determine the consequences of emotion regulation strategies used during music listening on hedonic outcomes, and to examine associations with emotional health and well-being.	Self-report (Experience sampling methodology/Questionnaire)	Using music to regulate recent emotions (response-focused strategies) achieved the greatest hedonic success but harmed emotional health and well-being. Strategies are chosen for desired outcomes based on mood and influenced by emotional health.
Shifriss et al. (2015) [34]	Israel	Quant	156	24–86	Jewish Israeli volunteer	o			1 h–3 h	Everyday	To examine beliefs about the impact of music on regulating a bad mood and to explore the differences in music choices (sad vs. happy) and their effects on mood regulation across different age groups.	Self-report (Questionnaire)	Participants in a bad mood who listen to music pay more attention to their emotions, use music for mood regulation more, and believe more in music’s power to influence their mood. Those preferring happy music in a bad mood tend to repair their mood better and believe more in music’s influence than those preferring sad music. Older participants favor happy music when feeling down.
Van den Tol et al. (2016) [35]	USA et al.	Quant	(1) 230 (2) 220	(1) *M* = 45.00 (2) *M* = 28.30	volunteered to participate via email invitation through various social research networks	o			NS	Everyday	To investigate why people listen to sad music when feeling sad, particularly focusing on its role in acceptance-based coping and consolation.	Self-report (Questionnaire)	People prefer sad music over happy music for consolation, despite generally liking happy music more. Sad music aids in accepting negative situations and emotions. These findings suggest SISM aids in coping by re-experiencing emotions.
White & Rickard (2016) [36]	Australia	Quant	32	18–28	Undergraduate psychology student cohort from Monash University	o			15 s	Experiment	To experimentally examine listeners’ capacity to regulate emotional responses induced by music.	Self-report (Questionnaire), ECG, SCL	Both “happy” and “sad” music increased self-reported emotions and reduced skin conductance and heart rate. Emotional responses were regulated for both music types, except for heart rate.
Baltazar et al. (2019) [37]	Sweden	Quant	35	19–44	Students and staff of Linköping University	o			3 min	Experiment	To determine the individual and relative impact of music and regulation strategies on stress reduction	Self-report (Questionnaire) SCL, EMG, EDA	Self-reported tension significantly reduced by both music and strategy.
Chang et al. (2020) [38]	USA	Quant	199	18–30	College students	o			NS	Everyday	To explore how deliberate music listening influences mood regulation, comparing music majors and non-music majors, and examining the relationship between positive music listening experiences and affect.	Self-report (Questionnaire)	Music majors scored significantly higher on the Positive Music Listening Experience Scale, positive affect and lower negative affect compared to non-music majors. Positive affect was significantly associated with most items related to positive music listening experiences.
Cheng (2020) [39]	China	Quant	92	*M* = 20.86	College students	o			NS	Experiment	To empirically analyze how different attributes of music, specifically speed and mode, affect emotional regulation in college students.	Self-report (Questionnaire)	Music speed has a significant impact on the emotions of college students, while the music mode does not.
Cook et al. (2019) [40]	USA	Quant	794	*M* = 21.68	Undergraduate students in the psychology subject pool of a large mid-western urban university	o			NS	Everyday	To investigate the relationship between music preferences and the use of music for emotion regulation among university students.	Self-report (Questionnaire)	Preferences for pop, rap/hip-hop, soul/funk, and electronica/dance music are positively associated with using music to increase emotional arousal.
Dingle & Fay (2017) [41]	Australia	Quant	60	18–25	Young adults	o			90 min	Experiment	To evaluate the effectiveness of “Tuned In,” a brief group intervention using music listening to teach young people emotional awareness and regulation skills.	Self-report (Questionnaire)	Participants in the Tuned In program experienced significant improvements in emotional awareness, clarity, and regulation compared to a control group.
Fancourt & Steptoe (2019) [42]	UK	Quant	2316	18+	Participants enrolled in the program Virtual Choir 5.0		o		NS	Experiment	To compare the experiences of social presence and the use of emotion regulation strategies (ERSs) between singers in virtual choirs (VCs) and live choirs.	Self-report (Questionnaire)	Participants in VCs reported a slightly greater feeling of social presence than those in live choirs but used overall fewer ERSs, avoidance strategies, and approach strategies, while making greater use of self-development strategies.
Groarke et al. (2020) [43]	Ireland	Quant	70	17–53	Undergraduate students with normal or corrected-to-normal vision and hearing who were over the age of 18 years were eligible to take part (or aged 17 with parental consent).	o			8 min	Experiment	To compare the effects of self-selected and researcher-selected music on induced negative affect (state anxiety and physiological arousal) and state mindfulness in young people.	Self-report (Questionnaire)	Study 1: Significantly greater anxiety reduction by music (both self-selected and researcher-selected). Study 2: Increased state mindfulness predicted lower anxiety after self-selected music listening.
Saarikallio et al. (2017) [44]	Sweden	Qual	55	15	Adolescents: attending high school	o			20 min	Experiment	To explore the affective dimension of adolescents’ musical relaxation by examining related affect regulation goals, strategies, and induction mechanisms.	Self-report (Interview)	Processing used both mechanisms; distraction and induction mainly used the musical mechanism. Musical distraction helped shift from negative to positive mood, while all methods equally supported positive emotion induction.
Shifriss et al. (2020) [45]	Israel	Quant	120	22–87	Jewish Israeli volunteer	o			1 min	Experiment	To investigate the association between age and the choice of happy music, moderated by the tendency to focus on emotions, and to examine the effects of music choice on mood regulation following a sad mood induction.	Self-report (Questionnaire)	Older adults who focus less on emotions prefer happy music after a sad mood. Those who focus more on emotions feel less negative after happy music, while those who focus less feel better after sad music.
Bachman et al. (2022) [46]	Israel	Quant	123	50+	Graduate students in the department of music at Bar-Ilan University.	o			NS	Everyday	To investigate the relationships between mindfulness, emotion regulation through music listening, and positive solitude (PS) in adults aged 50 and above.	Self-report (Questionnaire)	Significant positive associations between emotion regulation through music listening and PS, and between mindfulness and PS.
Berthold-Losleben et al. (2021) [47]	Netherlands	Quant	32	13	13 years of education level of school	o			15 min	Experiment	To investigate the effects of implicit regulation of negative emotions by positive stimuli on mood and related neuro-mechanisms and to explore its potential clinical relevance for treating psychiatric disorders with strong affective symptoms.	fMRI	Negative emotional state elicited by negative odours reduced by music training.
Carlson et al. (2021) [48]	Finland	Quant	432	18–77	Participants recruited through social media posts, University and professional e-mail lists and via both English and Finnish language press releases	o			NS	Everyday	To explore how people engaged with music during the first wave of the COVID-19 pandemic, specifically looking at music’s role in mood regulation and its relation to anxiety and worry.	Self-report (Questionnaire)	Positive correlations between participants’ use of mood for music regulation, their musical engagement, and their levels of anxiety and worry.
Carvalho et al. (2022) [49]	Portugal	Quant	48	19–33	Normal hearing and normal or correcte-to-normal vision	o			NS	Experiment	To investigate how music influences the implementation of emotion regulation strategies (distraction and reappraisal), particularly considering the moderating role of individual differences in musical sophistication and executive functioning.	Self-report (Questionnaire)	Participants with higher musical sophistication benefited from music during reappraisal but were impaired during distraction.
Coulthard et al. (2023) [50]	UK	Quant	80	5–7	Two primary schools in the East Midlands, UK.	o			4 min	Experiment	To examine whether listening to a happy song could counteract the effects of negative mood induction on snack food consumption in children, and to explore if parental feeding practices and child BMI would moderate these effects.	Self-report (Questionnaire)	No significant differences in the amount of snack food consumed between the happy music condition and the silent control condition.
Ferreri et al. (2021) [51]	Europe et al.	Quant	981	18	Online survey participants	o	o	o	NS	Everyday	To investigate changes in music-related habits due to the COVID-19 pandemic and to examine whether engagement in various music-related activities was associated with individual differences in musical reward, music perception, musical training, or emotional regulation strategies.	Self-report (Questionnaire)	The type of musical activity engaged in was linked to using music for stress regulation, addressing social interaction deficits, and cheering up, especially among those concerned about the virus and its consequences.
Garrido et al. (2022) [52]	Australia	Quant	24	13–25	Young people	o			NS	Everyday	To investigate the emotion-regulation strategies young people use when listening to music and the factors influencing the effectiveness of these strategies.	Self-report (Questionnaire)	Strategies Used: Mood-matching, mood-reducing, and mood-incongruent music to manage depression, anxiety, and tiredness. Intervening Factors: The severity of the prior mood, features of the music, and individual capacity for effective emotion regulation.
Gibbs & Egermann (2021) [53]	UK	Quant	570	18–84	Participants lived in the United Kingdom for at least the majority of the first lockdown and experienced ‘stay at home’ during the lockdown.	o			3 month	Everyday	To explore the nature of music-induced nostalgia during the first COVID-19 lockdown in the UK, analyze participants’ narratives and emotional responses to nostalgic music, and determine the impact of using nostalgic music listening as an emotion regulation strategy on wellbeing.	Self-report (Questionnaire)	Listening to nostalgic music during the lockdown positively impacted wellbeing by providing a sense of meaning and purpose.
Granot et al. (2021) [54]	11 countries: Argentina, Brazil, China, Colombia, Italy, Mexico, Netherlands, Norway, Spain, the UK, and USA,	Quant	5619	25+	Online survey participants	o	o	o	NS	Everyday	To investigate the role of music and personal or cultural variables in maintaining wellbeing during the COVID-19 crisis, focusing on how music helps achieve wellbeing goals during stress and social isolation.	Self-report (Questionnaire)	Music was the most effective activity for enjoyment, venting negative emotions, and self-connection.
Jakupčević et al. (2021) [55]	Croatia	Quant	252	18–49	Students of social sciences and humanities at the University of Split	o			NS	Everyday	To determine the relationship between mindfulness, absorption in music, and mood regulation through music in people with different musical tastes.	Self-report (Questionnaire)	Positive Correlations: Preferences for different music styles and absorption in music. Absorption in music and various strategies for regulating mood through music. Negative Correlations: Mindfulness and absorption in music. Mindfulness and most strategies for regulating mood through music.
Koehler et al. (2023) [56]	Germany, Switzerland, and Austria	Quant	428	*M* = 44.37	Online survey participants	o		o	NS	Everyday	To examine the bidirectional relationship between passive and active music engagement and affect over time, using a longitudinal approach.	Self-report (Questionnaire)	Negative Affect: More time spent with music listening (quantitative engagement) was associated with less negative affect at the next measurement. Positive Affect: No cross-lagged associations were found between music engagement and positive affect.
Larwood & Dingle (2022) [57]	UK	Quant	386	18–25	Residents of the Untied Kingdom who were recurited from profile.co	o			3 min	Experiment	To investigate how listener rumination and the eight BRECVEMA musical emotion mechanisms influence changes in sadness during listening to sad music in an induced sad state.	Self-report (Questionnaire)	Increase in Sadness by listening to a self-nominated sad song. High-rumination individuals were more likely to experience musical entrainment, select songs with conditioned responses and associated memories, and experience emotional contagion.
Li & Zheng (2021) [58]	China	Quant	21	23–31	College students	o			3 min	Experiment	To develop and evaluate a new method for regulating emotions using music, which addresses the limitations of traditional methods by employing different music processing techniques and stacked sparse auto-encoder neural networks.	EEG	Compared with complete music, the reconfigurable combined music was less time-consuming for emotional regulation (76.29% less), and the number of irrelevant emotional states was reduced by 69.92%. In terms of adaptability to different participants, the reconfigurable music improved the recognition rate of emotional states by 31.32%.
Liu et al. (2021) [59]	China	Quant	85	*M* = 20.69	Participants with right-handed, normal hearing and speech and normal or correcte-to-normal vision	o			3 min 20 s	Experiment	To explore the behavioral and neural correlates of mindfulness-based music listening for regulating induced negative emotions related to COVID-19, using the face–word Stroop task.	Self-report (Questionnaire), The Face-Word Stroop Task, EEG	Calm music and happy music effectively regulated young adults’ induced negative emotions, while young adults experienced more negative emotions when listening to sad music; the negative mood states at the post-induction phase inhibited the reaction of conflict control in face–word Stroop tasks, which manifested as lower accuracy (ACC) and slower reaction times (RTs). ERP results showed negative mood states elicited greater N2, N3, and LPC amplitudes and smaller P3 amplitudes.
Loureiro et al. (2024) [60]	Spain	Qual	17	15–16	4th grade Secondary Education students of a private school	o			NS	Experiment	To explore the functions of music listening in relation to emotion regulation and identity development in mid-adolescence.	Self-report (Interview)	Basic mood influence with upbeat music and complex emotional regulation with lyrics or melody. The latter was common in those with higher narrative meaning making, showing music’s role in emotional regulation and identity development during mid-adolescence.
Madden et al. (2023) [61]	USA et al.	Quant	421	*M* = 23	Musicians			o	NS		To investigate whether the emotions musicians desire during their practice are influenced by their personality traits and Mastery goal orientation (the desire to master musical and technical skills).	Self-report (Questionnaire)	Findings confirm a general hedonic principle underlying the emotions musicians desired in their musical practice. However, predicted by personality traits, musicians also sometimes sought to increase the intensity of unpleasant emotions.
Madden & Jabusch (2021) [62]	USA et al.	Quant	421	*M* = 21 (students) *M* = 31 (professionals)	Students and professional musicians of music institutions			o	last 2 weeks	Everyday	The aim of the study was to investigate emotion regulation behavior in the context of musical practice, specifically whether musicians adopt specific emotional stances to support their goal orientation and their beliefs about the functional impact of emotions.	Self-report (Questionnaire)	Musicians prefer affect-improvement strategies. Those valuing unpleasant emotions use affect-worsening strategies and focus on mastery goals. Some mastery-oriented musicians seek mixed emotions. Mastery-oriented are motivated by instrumental and hedonic benefits, while enjoyment-oriented prioritize hedonic benefits.
Maidhof et al. (2023) [63]	Germany	Quant	61	18–35	German, age 18–35 years, body mass index (BMI) of <30, no chronic physical disease, no hearing or severe visual impairment, no current psychological disorder, no medication intake or treatment with psychophysiological impact, smoking fewer than five cigarettes per week, no illegal drug consumption, no menstrua irregularities, no pregnancy, no breastfeeding, no profession associated with music.	o			NS	Experiment	To investigate the influence of music selection strategies (participant-selected vs. researcher-selected), gender, stimulus-induced emotions, and emotion regulation strategies on stress and mood responses.	Self-report (Questionnaire)	The study found no direct effect of music selection or gender on stress but noted gender-specific responses. Women had the strongest stress response and longest heart rate recovery with chosen music; Women showed more calmness variability and higher arousal with chosen music; Women used reappraisal, lowering stress, while men used suppression, increasing stress.
Ma’rof et al. (2023) [64]	UK & China	Quant	28	16–36	Participants in the UK and China	o			NS	Everyday	To examine the role of music in regulating emotions and to explore potential differences in music usage for emotion regulation between men and women in everyday life.	Self-report (Experience sampling methodology/ Questionnaire)	Relaxation was the most commonly used strategy for regulating emotions with music; Listening to music was an effective emotion regulation strategy, particularly for regulating happiness and peacefulness; Men were more likely to use music for active coping and to consider the type and content of music when selecting music; and music appeared to regulate the intensity of emotions similarly for both men and women, although men tended to report higher emotional intensity.
Martín et al. (2021) [65]	Spain	Quant	1377	41–60	University students	o			NS	Everyday	To investigate the relational influence of age on the frequency and form of music consumption, its use, and its value as a factor for emotional self-regulation during the COVID-19 pandemic confinement.	Self-report (Questionnaire)	Music was crucial for emotional support and alleviating loneliness during the pandemic, with a 56% increase in daily use for self-regulation. Music helped cope with anxiety, anguish, and depression, enhancing personal and social well-being across all ages.
Martínez-Castilla et al. (2021) [66]	Spain	Quant	507	18+	Participants of the online survey during the lockdown in Spain	o		o	3 month	Everyday	To analyze the impact of personal and context-related variables on the perceived efficacy of musical behaviors in fulfilling emotional wellbeing-related goals during the COVID-19 lockdown in Spain.	Self-report (Questionnaire)	The study found that personal variables, not COVID-19 context, affected the perceived efficacy of music. Young people and those with musical training saw the most benefit for well-being. Perceiving music as important was key to its efficacy.
Nwokenna et al. (2022) [67]	Nigeria	Quant	60	NS	Undergraduate students in music education			o	50 min	Experiment	To examine whether educational music intervention improves emotion regulation skills among first-year university music education students.	Self-report (Questionnaire)	Educational music intervention facilitated the development of emotional regulation skills in undergraduate music education students.
Park & Suh (2023) [68]	Korea	Quant	412	20–65	Online survey participants	o			NS	Everyday	To investigate the relationship between hardiness and Korean adults’ expectations for future life and to verify the multiple mediating effects of perceived stress, music listening for negative emotion regulation, and life satisfaction on that relationship.	Self-report (Questionnaire)	The study found hardiness positively correlated with using music for negative emotion regulation, life satisfaction, and future expectations, and negatively correlated with stress.
Randall et al. (2022) [69]	Finland	Quant	293	13–52	All of Finnish nationality. Any Finnish speaking person was eligible to participate if they used a mobile phone with the Android operat- ing system to listen to music.	o			5 min	Everyday	To determine the frequency with which listeners successfully reach their affect-regulatory goals through music listening on mobile phones, and to identify the predictors of this success.	Self-report (Experience sampling methodology/ Questionnaire)	Listeners successfully reached their affect-regulatory goals in less than half of the cases, with adults being more successful than adolescents.
Taruffi (2021) [70]	UK	Quant	26	*M* = 30.46	Participants recruited via Durham University student and staff mailing lists	o			5 min	Everyday	To explore the capacity of music to facilitate beneficial styles of mind-wandering and its experiential characteristics, using the experience sampling method to capture mind-wandering during personal music listening in everyday life.	Self-report (Experience sampling methodology/ Questionnaire)	Mind-wandering during music and non-music contexts was similar, with minor differences. Music-evoked emotions influenced thought valence, showing music’s effectiveness in regulating thoughts through emotion.
Tervaniemi et al. (2021) [71]	Finland	Quant	37	20–40	Adult healthy volunteers	o			10 min	Experiment	To compare music emotion ratings and their physiological correlates (specifically cortisol levels) when participants listen to music at home versus in the laboratory.	Self-report (Questionnaire), Saliva cortisol	Participants’ emotion ratings differed between home and lab settings, with lower cortisol levels at home. Both environments showed a decrease in cortisol levels after music listening, but the effect was consistent across settings.
Vidas et al. (2023) [72]	Australia	Quant	50	17–32	First-year international students	o			75 min	Experiment	To evaluate the effectiveness of the Tuned In program, an online group-based music listening intervention, for increasing emotion awareness, emotion regulation, and well-being in international students.	Self-report (Questionnaire)	Tuned In, even when delivered online, provides benefits for international students, suggesting that enjoyable programs that help develop emotion regulation skills.
Völker (2021) [73]	Germany	Quant	(1) 66 (2) 149	(1) *M* = 24.11 (2) *M* = 21.79	university students	o			(1) 2–10 min (2) 2:02–10:08	Experiment	To investigate the effects of self-selected music versus researcher-selected music on the induction of sadness and joy, considering the influences of perceptual and individual factors within a reciprocal-feedback model (RFM).	Self-report (Questionnaire)	Participant-chosen music strongly affected mood, with sadness and joy evoked by memory and contagion. Personal music increased cognitive activation, especially for joy.
Völker (2022) [74]	Germany	Quant	(1) 125 (2) 153	(1) *M* = 21.80 (2) *M* = 21.34	university students	o			(1) 10 min (2) NS	Experiment	To explore the indicators of spreading activation in the cognitive network and the emotion-inducing mechanisms of the BRECVEMA framework during music listening, and to examine how these factors are influenced by individual differences.	Self-report (Questionnaire)	Self-selected music, especially sad, enhances engagement and memory. These mechanisms aid in empathizing, systemizing, and reappraisal for emotion regulation. Sad music also links to habitual suppression and stronger conditioning/contagion.

## References

[1] Hanson-Abromeit D. (2015). A conceptual methodology to define the therapeutic function of music. Music Ther. Perspect..

[2] Juslin P.N., Sloboda J. (2011). Handbook of Music and Emotion: Theory, Research, Applications.

[3] Lundqvist L.O., Carlsson F., Hilmersson P., Juslin P.N. (2009). Emotional responses to music: Experience, expression, and physiology. Psychol. Music.

[4] Thompson W.F. (2015). Music, Thought, and Feeling: Understanding the Psychology of Music.

[5] Gabrielsson A. (2001). Emotion perceived and emotion felt: Same or different?. Music. Sci..

[6] Gross J.J., Thompson R.A. (2007). Emotion regulation: Conceptual foundations. Handbook of Emotion Regulation.

[7] Gross J.J. (1998). Sharpening the focus: Emotion regulation, arousal, and social competence. Psychol. Inq..

[8] Diener E. (1984). Subjective well-being. Psychol. Bull..

[9] Denollet J., Nyklìček I., Vingerhoets A.J. (2008). Emotion Regulation: Conceptual and Clinical Issues.

[10] Gross J.J. (2002). Emotion regulation: Affective, cognitive, and social consequences. Psychophysiology.

[11] Webb T.L., Miles E., Sheeran P. (2012). Dealing with feeling: A meta-analysis of the effectiveness of strategies derived from the process model of emotion regulation. Psychol. Bull..

[12] Bak S., Jeong Y., Yeu M., Jeong J. (2022). Brain–computer interface to predict impulse buying behavior using functional near-infrared spectroscopy. Sci. Rep..

[13] Jeong E., Ryu H., Shin J.H., Kwon G.H., Jo G., Lee J.Y. (2018). High oxygen exchange to music indicates auditory distractibility in acquired brain injury: An fNIRS study with a vector-based phase analysis. Sci. Rep..

[14] Zheng M., Lin H., Chen F. An fNIRS study on the effect of music style on cognitive activities. Proceedings of the 2020 42nd Annual International Conference of the IEEE Engineering in Medicine & Biology Society.

[15] Gallup G., Castelli J. (1989). The People’s Religion: American Faith in the 90′s.

[16] Sloboda J.A., Juslin P.N. (2001). Psychological perspectives on music and emotion. Music and Emotion: Theory and Research.

[17] Bogert B., Numminen-Kontti T., Gold B., Sams M., Numminen J., Burunat I., Lampinen J., Brattico E. (2016). Hidden sources of joy, fear, and sadness: Explicit versus implicit neural processing of musical emotions. Neuropsychologia.

[18] Juslin P.N., Liljeström S., Västfjäll D., Lundqvist L.O. (2010). How does music evoke emotions? Exploring the underlying mechanisms. Handbook of Music and Emotion: Theory, Research, Applications.

[19] Scherer K.R., Zentner M.R. (2001). Emotional effects of music: Production rules. Music Emot. Theory Res..

[20] Juslin P.N., Västfjäll D. (2008). Emotional responses to music: The need to consider underlying mechanisms. Behav. Brain Sci..

[21] Koelsch S. (2015). Music-evoked emotions: Principles, brain correlates, and implications for therapy. Ann. N. Y. Acad. Sci..

[22] Trost W.J., Labbé C., Grandjean D. (2017). Rhythmic entrainment as a musical affect induction mechanism. Neuropsychologia.

[23] Chong H.J., Kim B., Kim H.J. (2024). Analysis of music rationale and fidelity in music emotion regulation studies. J. Music Hum. Behav..

[24] Munn Z., Peters M.D.J., Stern C., Tufanaru C., McArthur A., Aromataris E. (2018). Systematic review or scoping review? Guidance for authors when choosing between a systematic or scoping review approach. BMC Med. Res. Methodol..

[25] Arksey H., O’Malley L. (2005). Scoping studies: Towards a methodological framework. Int. J. Soc. Res. Methodol..

[26] Peters M.D., Marnie C., Tricco A.C., Pollock D., Munn Z., Alexander L., Mcinerney P., Godfrey C.M., Khalil H. (2020). Updated methodological guidance for the conduct of scoping reviews. JBI Evid. Synth..

[27] Tricco A.C., Lillie E., Zarin W., O’Brien K.K., Colquhoun H., Levac D., Moher D., Peters M.D.J., Horsley T., Weeks L. (2018). PRISMA extension for scoping reviews (PRISMA-ScR): Checklist and explanation. Ann. Intern. Med..

[28] Carlson E., Saarikallio S., Toiviainen P., Bogert B., Kliuchko M., Brattico E. (2015). Maladaptive and adaptive emotion regulation through music: A behavioral and neuroimaging study of males and females. Front. Hum. Neurosci..

[29] Chin T., Rickard N.S. (2014). Emotion regulation strategy mediates both positive and negative relationships between music uses and well-being. Psychol. Music.

[30] Dingle G.A., Hodges J., Kunde A. (2016). Tuned in emotion regulation program using music listening: Effectiveness for adolescents in educational settings. Front. Psychol..

[31] Fernández-Sotos A., Fernández-Caballero A., Latorre J.M. (2016). Influence of tempo and rhythmic unit in musical emotion regulation. Front. Comput. Neurosci..

[32] Medrano L.A., Flores-Kanter E., Moretti L., Pereno G.L. (2016). Effects of induction of positive and negative emotional states on academic self-efficacy beliefs in college students. Psicol. Educ..

[33] Randall W.M., Rickard N.S., Vella-Brodrick D.A. (2014). Emotional outcomes of regulation strategies used during personal music listening: A mobile experience sampling study. Music. Sci..

[34] Shifriss R., Bodner E., Palgi Y. (2015). When you’re down and troubled: Views on the regulatory power of music. Psychol. Music.

[35] Van den Tol A.J., Edwards J., Heflick N.A. (2016). Sad music as a means for acceptance-based coping. Music. Sci..

[36] White E.L., Rickard N.S. (2016). Emotion response and regulation to “happy” and “sad” music stimuli: Partial synchronization of subjective and physiological responses. Music. Sci..

[37] Baltazar M., Västfjäll D., Asutay E., Koppel L., Saarikallio S. (2019). Is it me or the music? Stress reduction and the role of regulation strategies and music. Music Sci..

[38] Chang J., Lin P., Hoffman E. (2020). Music major, affects, and positive music listening experience. Psychol. Music.

[39] Cheng Z. (2020). Empirical analysis on the influence of music speed and music mode on the emotions of college students. Rev. Argent. Clínica Psicológica.

[40] Cook T., Roy A.R.K., Welker K.M. (2019). Music as an emotion regulation strategy: An examination of genres of music and their roles in emotion regulation. Psychol. Music.

[41] Dingle G.A., Fay C. (2017). Tuned in: The effectiveness for young adults of a group emotion regulation program using music listening. Psychol. Music.

[42] Fancourt D., Steptoe A. (2019). Present in body or just in mind: Differences in social presence and emotion regulation in live vs. virtual singing experiences. Front. Psychol..

[43] Groarke J.M., Groarke A., Hogan M.J., Costello L., Lynch D. (2020). Does listening to music regulate negative affect in a stressful situation? Examining the effects of self-selected and researcher-selected music using both silent and active controls. Applied psychology. Health Well-Being.

[44] Saarikallio S., Baltazar M., Västfjäll D. (2017). Adolescents’ musical relaxation: Understanding related affective processing. Nord. J. Music Ther..

[45] Shifriss R., Bodner E., Palgi Y. (2020). Don’t let me down: The effect of age and chosen music on mood is moderated by focus on emotions. J. Posit. Psychol..

[46] Bachman N., Palgi Y., Bodner E. (2022). Emotion regulation through music and mindfulness are associated with positive solitude differently at the second half of life. Int. J. Behav. Dev..

[47] Berthold-Losleben M., Papalini S., Habel U., Losleben K., Schneider F., Amunts K., Kohn N. (2021). A short-term musical training affects implicit emotion regulation only in behaviour but not in brain activity. BMC Neurosci..

[48] Carlson E., Wilson J., Baltazar M., Duman D., Peltola H.R., Toiviainen P., Saarikallio S. (2021). The role of music in everyday life during the first wave of the coronavirus pandemic: A mixed-methods exploratory Study. Front. Psychol..

[49] Carvalho M., Cera N., Silva S. (2022). The “ifs” and “hows” of the role of music on the implementation of emotional regulation strategies. Behav. Sci..

[50] Coulthard H., Van den Tol A.J., Jeffers S., Ryan S. (2023). Music as an alternative self-regulation strategy to snack foods following a negative mood induction in 5–7-year-old children: Interactions with parental use of food as a reward. Appetite.

[51] Ferreri L., Singer N., McPhee M., Ripollés P., Zatorre R.J., Mas-Herrero E. (2021). Engagement in music-related activities during the COVID-19 pandemic as a mirror of individual differences in musical reward and coping strategies. Front. Psychol..

[52] Garrido S., du Toit M., Meade T. (2022). Music listening and emotion regulation: Young people’s perspectives on strategies, outcomes, and intervening factors. Psychomusicol. Music. Mind Brain.

[53] Gibbs H., Egermann H. (2021). Music-evoked nostalgia and wellbeing during the United Kingdom COVID-19 pandemic: Content, subjective effects, and function. Front. Psychol..

[54] Granot R., Spitz D.H., Cherki B.R., Loui P., Timmers R., Schaefer R.S., Vuoskoski J.K., Cárdenas-Soler R., Soares-Quadros J.F., Li S. (2021). “Help! I need somebody”: Music as a global resource for obtaining wellbeing goals in times of crisis. Front. Psychol..

[55] Jakupčević K.K., Ercegovac I.R., Dobrota S. (2021). Music as a tool for mood regulation: The role of absorption vs. Mindfulness. Primenj. Psihol..

[56] Koehler F., Schäfer S.K., Lieb K., Wessa M. (2023). The interplay between music engagement and affect: A random-intercept cross-lagged panel analysis. Emotion.

[57] Larwood J.L., Dingle G.A. (2022). The effects of emotionally congruent sad music listening in young adults high in rumination. Psychol. Music.

[58] Li Y., Zheng W. (2021). Emotion recognition and regulation based on stacked sparse auto-encoder network and personalized reconfigurable music. Mathematics.

[59] Liu X., Liu Y., Shi H., Li L., Zheng M. (2021). Regulation of mindfulness-based music listening on negative emotions related to COVID-19: An ERP Study. Int. J. Environ. Res. Public Health.

[60] Loureiro C., van der Meulen K., del Barrio C. (2024). Why I listen to music: Emotion regulation and identity construction through music in mid-adolescence. Empiria Rev. Metodol. Cienc. Soc..

[61] Madden G.B., Herff S.A., Beveridge S., Jabusch H.C. (2023). Emotional cherry picking: The role of personality and goal orientation in selective emotion regulation for musical practice. Front. Psychol..

[62] Madden G.B., Jabusch H.C. (2021). Instrumental and hedonic motives for emotion regulation in musical practice. Front. Psychol..

[63] Maidhof R.M., Kappert M.B., Wuttke A., Schwerdtfeger A.R., Kreutz G., Nater U.M. (2023). Effects of participant-selected versus researcher-selected music on stress and mood—The role of gender. Psychoneuroendocrinology.

[64] Ma’rof A.A., Danhe Z., Zaremohzzabieh Z. (2023). Gender differences in the function of music for emotion regulation Development in everyday life: An experience sampling method study. Malays. J. Music.

[65] Martín J.C., Ortega-Sánchez D., Miguel I.N., Martín G.M.G. (2021). Music as a factor associated with emotional self-regulation: A study on its relationship to age during COVID-19 lockdown in Spain. Heliyon.

[66] Martínez-Castilla P., Gutiérrez-Blasco I.M., Spitz D.H., Granot R. (2021). The efficacy of music for emotional wellbeing during the COVID-19 lockdown in Spain: An analysis of personal and context-related variables. Front. Psychol..

[67] Nwokenna E.N., Sewagegn A.A., Falade T.A. (2022). Effect of educational music intervention on emotion regulation skills of first-year university music education students. Medicine.

[68] Park A., Suh K.H. (2023). Hardiness and Expectations for Future Life: The Roles of Perceived Stress, Music Listening for Negative Emotion Regulation, and Life Satisfaction. Behav. Sci..

[69] Randall W.M., Baltazar M., Saarikallio S. (2022). Success in reaching affect self-regulation goals through everyday music listening. J. New Music Res..

[70] Taruffi L. (2021). Mind-Wandering during Personal Music Listening in Everyday Life: Music-Evoked Emotions Predict Thought Valence. Int. J. Environ. Res. Public Health.

[71] Tervaniemi M., Makkonen T., Nie P. (2021). Psychological and Physiological Signatures of Music Listening in Different Listening Environments—An Exploratory Study. Brain Sci..

[72] Vidas D., Nelson N., Dingle G. (2013). Efficacy of the Tuned In music emotion regulation program in international university students. Psychol. Health.

[73] Völker J. (2021). Personalising music for more effective mood induction: Exploring activation, underlying mechanisms, emotional intelligence, and motives in mood regulation. Music. Sci..

[74] Völker J. (2022). Measuring emotional music experience: Spreading activation and BRECVEMA mechanisms. Psychol. Music.

[75] Dodge K.A. (1989). Coordinating responses to aversive stimuli: Introduction to a special section on the development of emotion regulation. Dev. Psychol..

[76] Walden T.A., Smith M.C. (1997). Emotion regulation. Motiv. Emot..

[77] Campos J.J., Frankel C.B., Camras L. (2004). On the nature of emotion regulation. Child Dev..

[78] Sheppes G., Levin Z. (2013). Emotion regulation choice: Selecting between cognitive regulation strategies to control emotion. Front. Hum. Neurosci..

[79] Kahle S., Miller J.G., Troxel N.R., Hastings P.D. (2021). The development of frustration regulation over early childhood: Links between attention diversion and parasympathetic activity. Emotion.

[80] Saarnl C., Crowley M. (2013). The development of emotion regulation: Effects on emotional state and expression. Emotions and the Family.

[81] Saarikallio S., Erkkilä J. (2007). The role of music in adolescents’ mood regulation. Psychol. Music.

[82] Baltazar M., Saarikallio S. (2016). Toward a better understanding and conceptualization of affect self-regulation through music: A critical, integrative literature review. Psychol. Music.

[83] Robb S.L., Carpenter J.S. (2009). A review of music-based intervention reporting in pediatrics. J. Health Psychol..

[84] Burns D.S. (2012). Theoretical rationale for music selection in oncology intervention research: An integrative review. J. Music Ther..

[85] Lee J.H. (2016). The effects of music on pain: A meta-analysis. J. Music Ther..

